# Monitoring Lipolysis by Sensing Breath Acetone down to Parts‐per‐Billion

**DOI:** 10.1002/smsc.202100004

**Published:** 2021-03-12

**Authors:** Ines C. Weber, Nina Derron, Karsten Königstein, Philipp A. Gerber, Andreas T. Güntner, Sotiris E. Pratsinis

**Affiliations:** ^1^ Particle Technology Laboratory Department of Mechanical and Process Engineering ETH Zurich CH-8092 Zurich Switzerland; ^2^ Department of Endocrinology, Diabetology, and Clinical Nutrition University Hospital Zurich (USZ) and University of Zurich (UZH) CH-8091 Zurich Switzerland; ^3^ Division Sports and Exercise Medicine Department of Sport, Exercise and Health University of Basel CH-4052 Basel Switzerland

**Keywords:** breath acetone sensors, end‐tidal acetone, mobile health, monitoring lipolysis

## Abstract

Mobile health technologies can provide information routinely and on demand to manage metabolic diseases (e.g., diabetes and obesity) and optimize their treatment (e.g., exercise or dieting). Most promising is breath acetone monitoring to track lipolysis and complement standard glucose monitoring. Yet, accurate quantification of acetone down to parts‐per‐billion (ppb) is difficult with compact and mobile devices in the presence of interferants at comparable or higher concentrations. Herein, a low‐cost detector that quantifies end‐tidal acetone during exercise and rest is presented with excellent bias (25 ppb) and unprecedented precision (169 ppb) in 146 breath samples. It combines a flame‐made Pt/Al_2_O_3_ catalyst with a chemoresistive Si/WO_3_ sensor. The detector is robust against orders of magnitude higher ethanol concentrations from disinfection and exercise‐driven endogenous breath isoprene ones, as validated by mass spectrometry. This detector accurately tracks the individual lipolysis dynamics in all volunteers, as confirmed by blood ketone measurements. It can be integrated readily into handheld devices for personalized metabolic assessment at home, in gyms, and clinics.

## Graphical abstract

A compact and low‐cost acetone detector tracks lipolysis from exhaled breath during exercise and rest, in excellent agreement with mass spectrometry and blood ketone measurements. It exhibits high robustness to endogenous isoprene and background ethanol by screening a Si/WO_3_ sensor with a selective catalytic Pt/Al_2_O_3_ filter. Hence, this detector is promising for personalized and non‐invasive metabolic assessment.

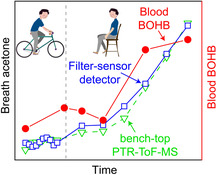

## 1 Introduction

The metabolic syndrome^[^ ^1^ ^]^ (e.g., abdominal obesity and increased blood triglycerides) is a global epidemic associated with cardiovascular diseases, diabetes, non‐alcoholic fatty liver disease, and cancer, to name a few.^[^ ^2^ ^]^ Despite growing public awareness and an abundance of preventive and therapeutic interventions to tackle the consequences of obesity (13% of world's population^[^ ^3^ ^]^), negative health impacts (e.g., 4 million deaths and 120 million disability‐adjusted life years globally^[^ ^4^ ^]^ in 2015) still prevail even among adolescents.^[^ ^5^ ^]^ Moreover, obesity and related diseases are expected to increase further with projected expenses of about 57 billion USD per year by 2030 in the USA alone.^[^ ^6^ ^]^ Miniaturized mobile health (mHealth) devices^[^ ^7^ ^]^ are most desirable to enable on‐demand metabolic monitoring of physical activity and nutrition while abnormalities can also be identified at an early stage. Such devices provide user feedback at the point‐of‐care^[^ ^8^ ^]^ and facilitate personalized treatment and prevention of metabolic diseases.

Since the first^[^ ^9^ ^]^ continuous blood glucose monitoring (CGM) device was approved by the Food and Drug Administration in 1999, the management of diabetes type‐1 has been significantly improved.^[^ ^10^ ^]^ Also, this device has been explored for type‐2 diabetes^[^ ^11^ ^]^ and gestational diabetes mellitus in pregnant women.^[^ ^12^ ^]^ The CGM was enabled through advances in electrochemical sensors^[^ ^13^ ^]^ that paved the way for a multitude of follow‐up innovations, including automated insulin delivery (e.g., glucose‐responsive insulin delivery patches^[^ ^14^ ^]^ and artificial pancreas systems^[^ ^15^ ^]^) and wearable contact lens biosensors.^[^ ^16^ ^]^ Yet, missing are complementary tools that monitor lipolysis and track its enhancement (e.g., to treat obesity through exercise and dieting) or reduction (to prevent/treat ketoacidosis in diabetes) to guide lifestyle changes. Currently, lipolysis can be assessed in vivo either through blood ketones, using tracer‐labeled glycerol in blood plasma or microdialysis techniques in adipose tissue, all being invasive techniques requiring trained personnel.^[^ ^17^ ^]^ However, the possibility of point‐of‐care self‐monitoring would help understand patient's lipolysis dynamics as well as guide therapeutic action.

Breath acetone is most promising as a biomarker for tracking metabolic changes.^[^ ^18^ ^]^ It originates from lipolysis where fatty acids undergo hepatic β‐oxidation to acetyl coenzyme A and acetoacetate, that is degraded into volatile acetone and β‐hydroxybutyrate (BOHB).^[^ ^19^ ^]^ Being volatile, acetone can be detected non‐invasively, routinely, and online^[^ ^18^ ^]^ by breath analysis^[^ ^20^ ^]^ (as established in clinics already for nitric oxide in detection of airway inflammation^[^ ^21^ ^]^), with high user tolerance even in a non‐diseased population.

Compact,^[^ ^22^ ^]^ low‐cost, and highly sensitive^[^ ^23^ ^]^ acetone sensors already exist even commercially (e.g., LEVL, Ketonix, Keyto, Lexico Health Keto Breath Analyzer, ACE KETOSCAN mini). They are usually based on solid‐state^[^ ^24^ ^]^ chemoresistive^[^ ^25^ ^]^ (metal oxides^[^ ^26^ ^]^ or carbon‐based hybrids^[^ ^27^ ^]^), electrochemical^[^ ^28^ ^]^ or optical^[^ ^18^ ^]^ sensors, or arrays.^[^ ^29^ ^]^ Despite extensive research^[^ ^24^ ^]^ since 1984, such acetone sensors still have not been established for routine healthcare monitoring,^[^ ^30^ ^]^ as they usually fall short on accuracy,^[^ ^31^ ^]^ mostly due to lack of acetone selectivity. For instance, breath acetone ranges between 0.7 and 1 ppm during moderate and constant‐load exercise.^[^ ^32^ ^]^ So, fine concentration differences need to be tracked to indicate and reveal anaerobic thresholds (i.e., breath acetone increase^[^ ^33^ ^]^ of 25%) or to distinguish cardiorespiratory fitness.^[^ ^34^ ^]^ Similar small changes in breath acetone concentration should be recognized when assessing the effectiveness of intermittent fasting (i.e., BOHB increase of 60% after four weeks of alternate day fasting^[^ ^35^ ^]^), whereas periodically elevated BOHB levels (i.e., 1.5 mmol L^−1^ compared with basal 0.12 mmol L^−1^) have anti‐aging and cardioprotective effects in mice.^[^ ^36^ ^]^ Today's state‐of‐the‐art acetone sensors can hardly resolve these differences having precision not better than 0.6 ppm (**Table** **1**). This is mostly associated with their weak acetone selectivity, as critical interferants can be orders of magnitude higher such as ethanol (e.g., >100 ppm in gym or hospital air from hand disinfection^[^ ^37^ ^]^) or H_2_ (e.g., 5.5 ppm in exhaled breath^[^ ^38^ ^]^). Only in extreme cases, such as ketogenic diets^[^ ^39^ ^]^ or diabetic ketoacidosis,^[^ ^40^ ^]^ when acetone concentrations exceed 50 ppm, these interferants become less problematic.

**Table 1:** Bias and precision of portable acetone detectors with available data for human breath. GC‐FID: gas chromatography‐flame ionization detector, SIFT‐MS: selected‐ion flow‐tube mass spectrometry, GC‐MS: gas chromatography‐mass spectrometry, GC: gas chromatography, and PTR‐ToF‐MS: proton‐transfer‐reaction time‐of‐flight mass spectrometry

Type	Name	Bias [ppb]	Precision [ppb]	# breath samples [–]	Validation method	Ref
	LEVL	1000	1000	–	–	[69]
Optical	Adsorption column	1274	3237	66	GC‐FID	[18]
	Colorimetric	12	626	45	SIFT‐MS	[70]
Electro‐chemical	Enzymatic sensor	1855	3052	38	GC‐MS	[28]
Chemoresistive						
Array	Pt/WO_3_ and SnO_2_	132	645	238	GC	[22]
Single sensor	Si/WO_3_	271	442	146	PTR‐ToF‐MS	This work
With filter	Pt/Al_2_O_3_–Si/WO_3_	25	169	146	PTR‐ToF‐MS	

**Figure 1:**
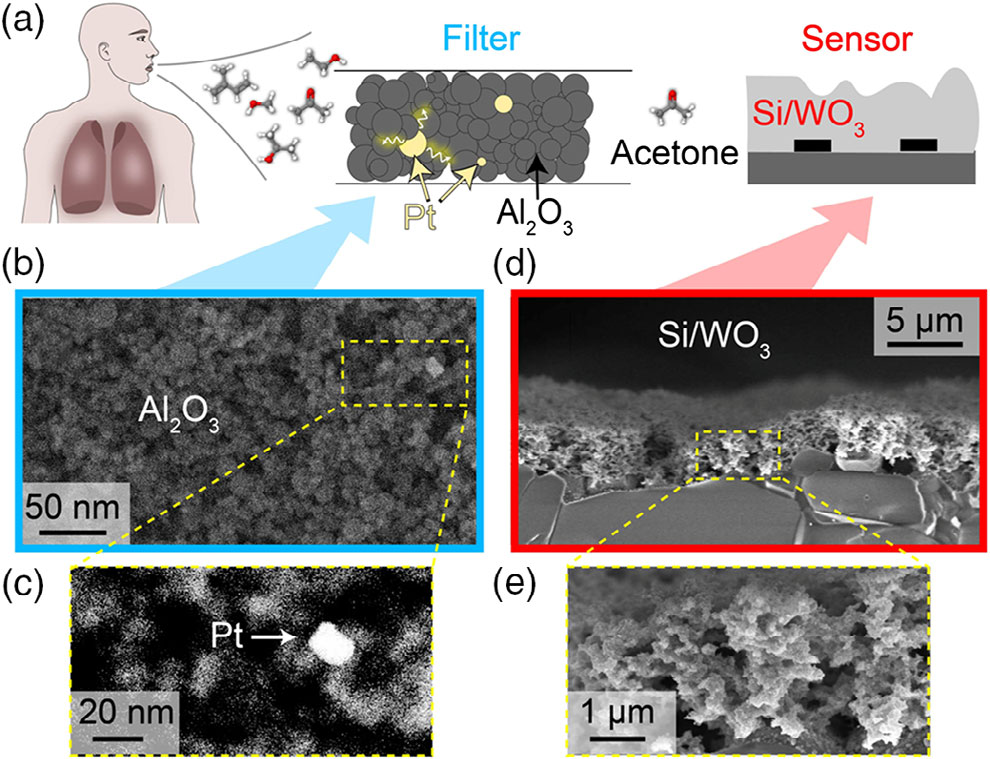
a) Schematic of the filter‐sensor detector concept: Breath molecules reach the catalytic Pt/Al_2_O_3_ filter where interferants are removed by chemical reaction. Only acetone reaches the Si/WO_3_ sensor where it is quantified. The Pt/Al_2_O_3_ nanoparticles are imaged with b) a secondary electron detector and c) a high angle annular dark field detector in the yellow‐framed area (in (b)) for better visibility of the Pt clusters (bright in (c)). d) Scanning electron microscopy image (cross section) of the highly porous flame‐aerosol‐deposited Si/WO_3_ sensor film, together with a higher magnification in (e).

Here, accurate breath acetone monitoring during physical activity and rest is reported with a low‐cost detector. It is based on a compact assembly^[^ ^41^ ^]^ of a flame‐made^[^ ^42^ ^]^ Pt/Al_2_O_3_ catalytic filter and a chemoresistive Si/WO_3_ gas sensor^[^ ^23^ ^]^ that quantifies acetone selectively at high relative humidity (RH; **Figure** **1**a), as proved with laboratory gas mixtures.^[^ ^41^ ^]^ This detector (Pt/Al_2_O_3_–Si/WO_3_) was tested on 146 end‐tidal breath samples from a cardiorespiratory fitness‐adjusted^[^ ^34^ ^]^ exercise protocol and subsequent 3 h rest.^[^ ^43^ ^]^ Hence, the detector's capacity to monitor lipolysis was assessed in a demanding application, where small acetone changes (e.g., <0.5 ppm^[^ ^32^ ^]^) need to be quantified from short (i.e., 5 s) exhalations, in the presence of endogenous compounds (e.g., isoprene up to 0.44 ppm) and background ethanol (up to 3.2 ppm) from disinfectants. This was quite a challenge for the Si/WO_3_ sensor alone as had neither been considered in laboratory tests^[^ ^23^ ^]^ nor offline^[^ ^44^ ^]^ and online^[^ ^45^ ^]^ breath studies during exercise^[^ ^43^ ^]^ and diet.^[^ ^39^ ^]^ Finally, a Bland–Altman analysis^[^ ^46^ ^]^ was performed to compare the device's bias and precision to bench‐top PTR‐ToF‐MS and state‐of‐the‐art breath acetone detectors.

## 2 Results and Discussion

### 2.1 Selective Breath Acetone Quantification

The detector concept is schematically shown in Figure 1a with a photograph of the filter, sensor, and assembled detector being provided in Figure S1, Supporting Information. In principle, exhaled breath with its more than 800 volatiles^[^ ^47^ ^]^ is pre‐screened by a catalytic filter that continuously converts critical interferants (e.g., isoprene, methanol, ethanol, and 2‐propanol) to sensor‐inert species. The acetone remains largely intact and is selectively detected by the downstream sensor. The catalytic filter consists of flame‐made^[^ ^42^ ^]^ Pt/Al_2_O_3_ nanoparticles due to their preferential conversion of confounding alcohols,^[^ ^48^ ^]^ aromatics, aldehydes, hydrocarbons,^[^ ^49^ ^]^ H_2_ and CO on surface‐adsorbed hydroxyl groups. The nanoparticles contain 0.2 mol% Pt that promotes the catalyst reactivity with these confounders at 135 °C. In contrast, acetone is converted by coordinative binding^[^ ^50^ ^]^ to Lewis acid sites.^[^ ^51^ ^]^ However, these are blocked here (i.e., due to high humidity of exhaled breath) by water molecules that dissociate on such sites.^[^ ^52^ ^]^ This results in unmet acetone selectivity, as demonstrated with laboratory gas mixtures^[^ ^41^ ^]^ and evaluated here for more challenging human breath.

These nanoparticles are visualized in Figure 1b, where mostly Al_2_O_3_ particles (<20 nm) are observed. With a high angle annular dark field detector (Figure 1c), the Pt clusters appear brighter than Al due to their higher scattering potential. The chemoresistive sensor consists of Si‐containing ε‐WO_3_ (Si/WO_3_) that exhibits good acetone selectivity over ethanol.^[^ ^23^ ^]^ Such Si/WO_3_ particles were made by flame spray pyrolysis (FSP) and directly deposited as highly porous (Figure 1d,e) and 3.7 ± 0.6 μm thick (Figure S2, Supporting Information) films, enabling acetone detection down to 20 parts‐per‐billion (ppb) at 90% RH.

**Figure 2:**
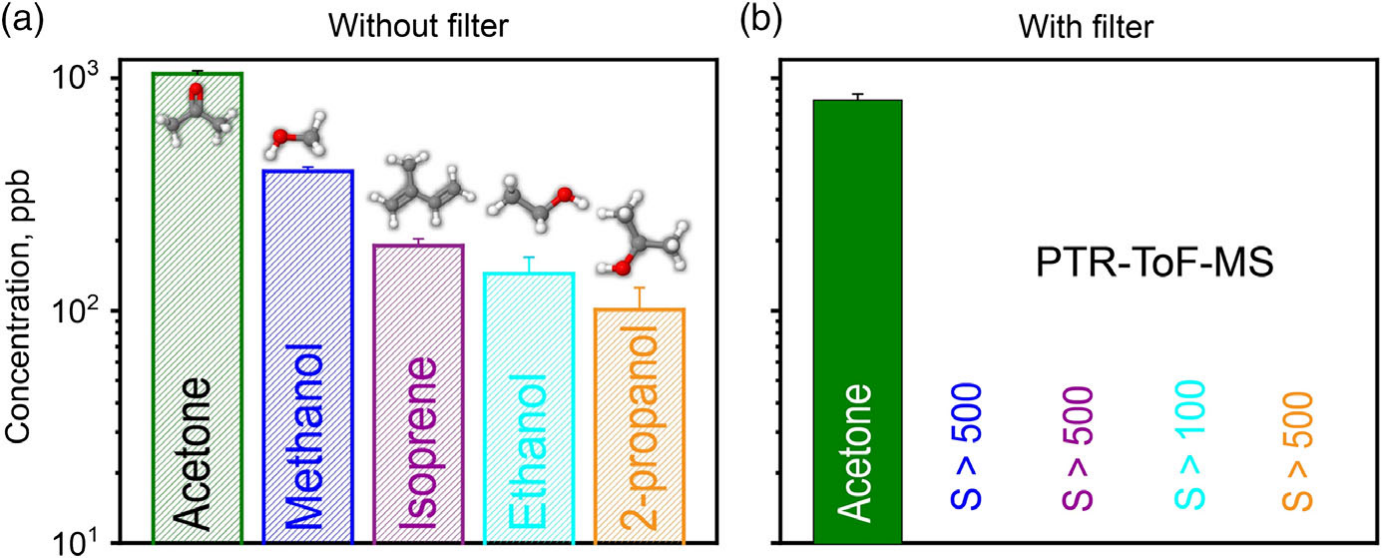
Breath acetone, methanol, isoprene, ethanol, and 2‐propanol concentrations a) without and b) with the preceding Pt/Al_2_O_3_ filter (the acetone selectivity is indicated for each species in (b)), as measured by bench‐top PTR‐ToF‐MS. Columns indicate average concentrations and error bars the standard deviations in three consecutive exhalations (*n* = 3) of volunteer #2.

As a first step, the filter was tested on three end‐tidal exhalations after overnight fasting (volunteer #2) that contained acetone (average ± standard deviation (*σ*): 1044 ± 31 ppb), methanol (397 ± 17 ppb), isoprene (190 ± 13 ppb), ethanol (144 ± 26 ppb), and isopropanol (101 ± 24 ppb), as quantified by high‐resolution PTR‐ToF‐MS (**Figure** **2**a). These concentrations are consistent with similarly sampled ones from 30 healthy humans,^[^ ^53^ ^]^ for example, for acetone (ranging from 148 to 2744 ppb) and isopropanol (from 0 to 135 ppb). Most importantly, the filter completely removed all these breath compounds (i.e., <5 ppb, Figure 2b) except for acetone (i.e., loss of 23%), resulting in its excellent selectivity (*S* > 100, Figure 2b). This is in line with breath and laboratory gas mixtures for acetone, methanol, isoprene, and ethanol^[^ ^41^ ^]^ and also confirmed here for isopropanol. This outstanding acetone selectivity is also preserved when the filter is coupled to the Si/WO_3_ sensor, because almost identical (4% deviation) responses are obtained for 1000 ppb acetone at 90% RH in synthetic air and for a breath sample containing 1018 ppb acetone at ≈97% RH^[^ ^54^ ^]^ (Figure S3a, Supporting Information). This is superior to state‐of‐the‐art acetone sensors that feature lower selectivity, for example, with respect to ethanol (i.e., 12.5 with an optical La_2_O_3_ sensor^[^ ^55^ ^]^), isoprene (i.e., 3.6 with Al‐doped ZnO^[^ ^56^ ^]^), or methanol (i.e., 4.7 with Co‐doped ZnO nanofibers^[^ ^57^ ^]^). Therefore, breath acetone can be simply quantified by comparing the detector breath response to that of the calibrated acetone standard in synthetic air at 90% RH (Figure S3b, Supporting Information).

### 2.2 Breath Acetone Monitoring during Exercise and Rest

**Figure 3:**
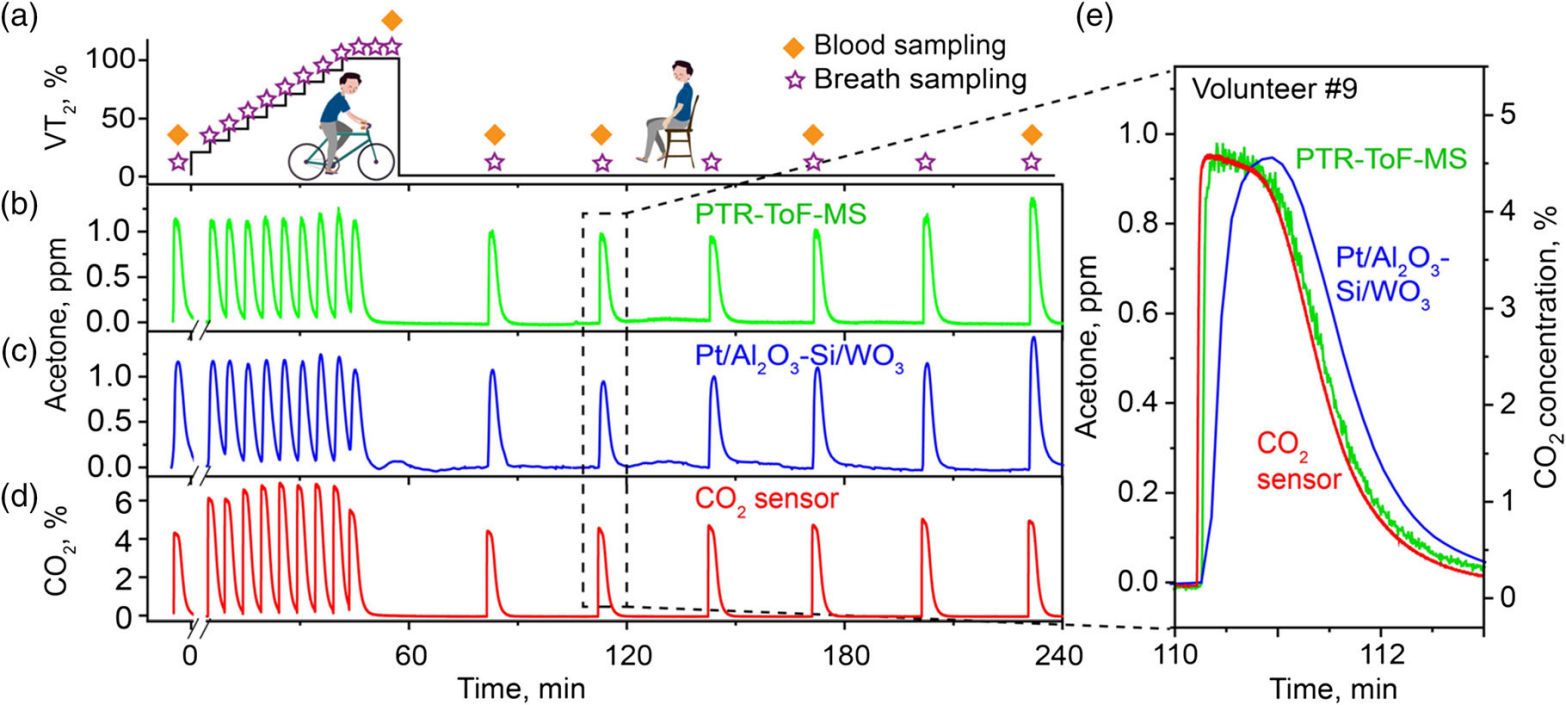
a) The protocol was standardized to the cardiorespiratory fitness^[^ ^34^ ^]^ comprising a ramped exercise (0 ≤ *t* < 60 min) until the individual second ventilatory threshold (VT_2_) and a resting phase (*t* > 60 min). Breath (stars) and blood (diamonds) sampling is indicated. Breath acetone detected by b) the PTR‐ToF‐MS and c) the Si/WO_3_ sensor with Pt/Al_2_O_3_ filter exemplarily for volunteer #9. d) Simultaneous CO_2_ measurement to confirm correct end‐tidal breath sampling (i.e., CO_2_ > 3%^[^ ^60^ ^]^). e) Magnification of an exemplary exhalation (at about 110 min, see dashed box in (b–d)).

Next, the detector was tested with volunteers during and after an exhaustive and cardiorespiratory fitness‐adapted^[^ ^34^ ^]^ exercise protocol (**Figure** **3**a) where breath (stars) and blood (diamonds) sampling are indicated. The responses with the PTR‐ToF‐MS (Figure 3b), the acetone detector (Figure 3c), and a commercial CO_2_ sensor (Figure 3d) are shown exemplarily for volunteer #9. In all exhalations, the detector indicates breath acetone with identical resolution to bench‐top PTR‐ToF‐MS and in sync with a commercial CO_2_ sensor, demonstrating its multiuse capacity to monitor metabolic breath species even with fast time resolution.

Figure 3e shows a juxtaposition of the PTR‐ToF‐MS (green), detector (blue) for acetone, and CO_2_ sensor (red) responses exemplarily for the second sampled pulse during resting (dashed box in Figure 3b–d). Note that all volunteers exhaled completely (within 5 s) into the end‐tidal breath sampler.^[^ ^58^ ^]^ Therein, breath from the upper airways is separated from the end‐tidal portion^[^ ^59^ ^]^ (as confirmed by the final CO_2_ concentrations of 4.2–6.6 vol% being higher than >3%^[^ ^43^, ^58^, ^60^ ^]^), which is buffered for analysis, because it best reflects the blood chemistry and, thus, lipolysis. When analyzed by PTR‐ToF‐MS, breath acetone increases rapidly (response time of 8 s) up to the end‐tidal concentration of 945 ppb (buffered for 50 s). Note that breath acetone and CO_2_ are not correlated (Figure S4, Supporting Information), given CO_2_'s different origin from intracellular metabolism reflecting overall energy consumption rather than lipolysis.^[^ ^61^ ^]^ The Si/WO_3_ sensor screened by the Pt/Al_2_O_3_ filter measures an almost identical acetone level (i.e., 947 ppb) with longer though sufficiently fast response time (i.e., 35 s, in line with 28 s of the Si/WO_3_ sensor alone^[^ ^45^ ^]^). In the following, only the end‐tidal breath acetone levels are discussed.

**Figure 4:**
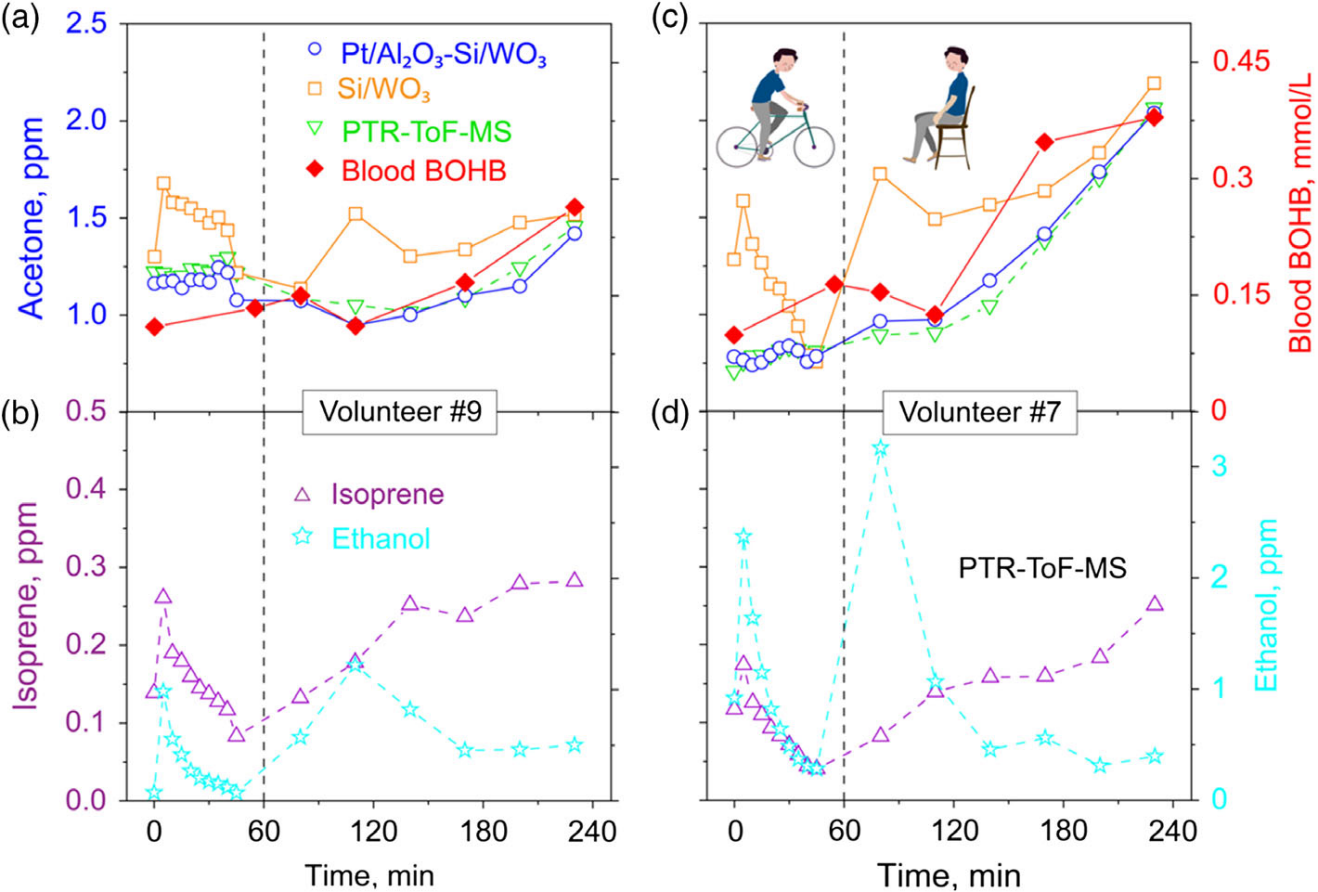
Breath acetone concentrations by the detector (Pt/Al_2_O_3_–Si/WO_3_, circles), the sensor without filter (Si/WO_3_, squares), and the PTR‐ToF‐MS (inverted triangles) along with blood BOHB concentrations (diamonds) from volunteers a) #9 and c) #7 during exercise (*t* < 60 min, dashed line) and rest (*t* > 60 min) from single exhalations. b,d) Corresponding breath isoprene (triangles) and ethanol (stars) concentrations by PTR‐ToF‐MS are provided for these volunteers. Note the different ordinate scales. Data for all volunteers are presented in Figure S5, Supporting Information.

**Figure** **4** shows breath acetone concentrations measured by the sensor without (squares) and with (circles) the filter and PTR‐ToF‐MS (inverted triangles), along with blood BOHB concentrations (diamonds) for volunteers #9 (Figure 4a) and #7 (Figure 4c). Their isoprene (triangles) and ethanol (stars) concentrations are shown in Figure 4b,d, respectively. Note that breath and blood data for all volunteers are shown in Figure S5, Supporting Information, whereas their physiological ones are provided in Table S1, Supporting Information.

For volunteer #9, acetone concentrations remain rather constant during exercise (i.e., 1.23 ± 0.03 ppm). They start increasing only after 3 h of resting (i.e., 1.46 ppm). This small increase indicates weak exercise‐related lipolysis activation, in agreement with the literature^[^ ^34^ ^]^ for such subjects with low cardiorespiratory fitness (i.e., male subject with VO_2peak_ < 40 mL kg^−1^ min^−^ ^1^,^[^ ^62^ ^]^ Table S1, Supporting Information). This is consistent with his BOHB measurements (diamonds, Figure 4a) that hardly change during exercise and increase only little during resting. In contrast, high‐fit volunteer #7 (female subject with VO_2peak_ > 34 mL kg^−1^ min^−1^,^[^ ^62^ ^]^ Table S1, Supporting Information) shows a distinct acetone increase during exercise (i.e., from 0.71 to 0.81 ppm) and reaches 2.06 ppm after 3 h of rest. Hence, her lipolysis was stimulated more effectively, in line with the literature,^[^ ^34^ ^]^ and also confirmed by a quite similar increase in her blood BOHB (Figure 4c). This was also observed for the other volunteers (Figure S5, Supporting Information) with some (e.g., volunteers #2 and #8) entering even nutritional ketosis (i.e., BOHB 0.5–3 mmol L^−1^^[^ ^63^ ^]^) toward the end of resting, as was observed also during 36 h ketogenic fasting in healthy subjects.^[^ ^39^ ^]^

The Pt/Al_2_O_3_–Si/WO_3_ detector (Figure 4a,c, circles) accurately tracks the breath acetone measured by PTR‐ToF‐MS (inverted triangles) for both volunteers, reflecting well the blood BOHB (diamonds) and can, thus, be applied to monitor individual lipolysis. This is due to its excellent robustness to endogenous (e.g., isoprene) and background (e.g., ethanol) interferants. In fact, isoprene spikes (within 2 min^[^ ^32^ ^]^) at the onset of muscle activity and decreases thereafter, as also observed here for all volunteers (Figure 4b,d, and Figure S5, triangles, Supporting Information). This does not affect the detector's acetone measurements. Furthermore, ethanol concentrations up to 3.2 ppm (Figure 4b,d, stars) from hand disinfection usually applied prior to blood sampling (e.g., before *t* = 5, 80, and 110 min) did not interfere the detector, as also consistently observed for the other volunteers (Figure S5, Supporting Information). Actually, even up to 100 ppm ethanol is removed effectively by the filter (≥98%, Figure S6, Supporting Information), whereas its performance to convert higher concentrations (e.g., up to 180 ppm) potentially present in gyms and hospitals^[^ ^37^ ^]^ can be further improved by adding a modular ZnO catalytic filter.^[^ ^64^ ^]^ Also, other common sensor interferants in breath or background air such as H_2_ or CO seem not to affect the sensor, in agreement with previous measurements^[^ ^41^ ^]^ with gas standards, where up to 100 ppm H_2_ and 50 ppm CO were removed completely with this filter.

It is worth noting that without the Pt/Al_2_O_3_ filter, the Si/WO_3_ sensor is interfered by isoprene showing a similar “acetone” spike after 5 min of exercise for almost all volunteers (Figure 4a,c, and Figure S5, squares, Supporting Information) in stark contrast to the PTR‐ToF‐MS (inverted triangles). This can lead to significant errors. For example, a 110% maximum acetone overprediction for volunteer #7 at *t* = 5 min is indicated (Figure 4c) when both isoprene and ethanol peak at the same time (Figure 4d). Note that other state‐of‐the‐art acetone sensors had rarely been tested with isoprene and most likely had been interfered by it as well (e.g., Al‐doped ZnO^[^ ^56^ ^]^). In fact, when the same Si/WO_3_ sensor was tested on a subject,^[^ ^45^ ^]^ its response increased upon physical activity (Figure 6b in the previous study^[^ ^45^ ^]^) despite constant acetone levels by PTR‐ToF‐MS. This suggests isoprene sensitivity, as confirmed later with laboratory standards.^[^ ^41^ ^]^ Similarly, also the ethanol interferes with the Si/WO_3_ sensor alone, as is observed for all volunteers (Figure 4a,c, and Figure S5, Supporting Information). For example, this leads to a 90% acetone overprediction for volunteer #7 at *t* = 80 min (Figure 4c). This is most problematic in applications where background ethanol concentrations cannot be prevented (e.g., gyms and hospitals).

**Figure 5:**
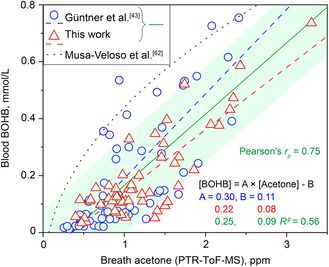
Comparison of breath acetone and blood BOHB concentrations (triangles, *n* = 53 samples), together with previous measurements^[^ ^43^ ^]^ during and after exercise (circles, *n* = 60). Note that one blood sample of this work (volunteer #8) was not analyzed due to blood clotting. Breath acetone and blood BOHB were determined by PTR‐ToF‐MS and venous blood analysis, respectively. The linear fits (blue (Güntner et al.^[^ ^43^ ^]^), red (this work) dashed lines, and green (all data) solid line with ± σ (shade)) and respective equations are indicated together with the coefficient of determination (*R* ^2^) and Pearson's correlation coefficient (*r*_p_) for all data points. An exponential fit proposed by Musa‐Veloso et al.^[^ ^68^ ^]^ for ketogenic diets (dotted line) is shown as well.

Finally, the correlation between breath acetone (measured by PTR‐ToF‐MS) and blood BOHB is shown in **Figure** **5** with the present data (triangles, *n* = 53 samples) along with those of Güntner et al.^[^ ^43^ ^]^ collected during and after exercise (circles, *n* = 60 samples), however, with a different cycling protocol (i.e., constant load at moderate intensity). There is good agreement between both data sets, and the overall correlation coefficients between breath acetone and blood BOHB are *r*_p_ = 0.75 (Pearson's, *p* < 0.05) and *r*_s_ = 0.74 (Spearman's, *p* < 0.05). This is in line with previous studies that as well correlated these parameters, for instance, in type‐1 diabetics after overnight fasting (*r*_p_ = 0.57^[^ ^65^ ^]^), during the day (morning and afternoon, 0.93^[^ ^66^ ^]^) or ketoacidosis (0.82^[^ ^67^ ^]^) and in healthy subjects during ketogenic diets (0.78^[^ ^39^ ^]^) for different concentration ranges. Linear relationships between blood BOHB and breath acetone for the present data (red dashed line, Figure 5), Güntner et al.,^[^ ^43^ ^]^ (blue dashed line), and the combined data set (green solid line) are indicated together with the equations of those fits and the coefficient of determination (*R* ^2^ = 0.56). Note that an exponential fit was suggested by Musa‐Veloso et al. (dotted line, Figure 5, 0–8 ppm breath acetone; 0–1.5 mmol L^−1^ BOHB^[^ ^68^ ^]^), however, for a different lipolysis stimulus (i.e., ketogenic diet).

### 2.3 Detector Bias and Precision

**Figure 6:**
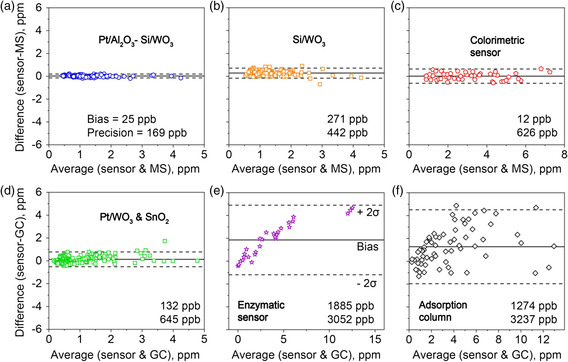
Bland–Altman^[^ ^46^ ^]^ analysis: Difference between breath acetone concentrations by the PTR‐ToF‐MS and the Si/WO_3_ sensor a) with and b) without the Pt/Al_2_O_3_ filter as a function of their average for *n* = 146 samples. Difference between state‐of‐the art sensors and SIFT‐MS or GC for c) a hydroxylamine sulfate colorimetric sensor,^[^ ^70^ ^]^ d) a Pt/WO_3_ and SnO_2_ sensor array,^[^ ^22^ ^]^ e) an enzymatic sensor,^[^ ^28^ ^]^ and f) a sensor based on an adsorption column.^[^ ^18^ ^]^ Solid lines indicate the bias (mean of the difference); dashed lines indicate the limit of agreement (bias ± 2*σ*). The biases and precisions (2*σ*) are quantified. Note the different testing protocols, thus different acetone concentrations ranges for the measurements, as specified in some of the cited literature.

**Figure** **6**a shows the Bland–Altman^[^ ^46^ ^]^ analysis of the Pt/Al_2_O_3_–Si/WO_3_ detector compared with the bench‐top PTR‐ToF‐MS for all nine volunteers (i.e., 146 breath samples). Excellent agreement is obtained, featuring a bias and precision of 25 and 169 ppb, respectively. This detector is a clear improvement over the Si/WO_3_ sensor alone (Figure 6b, 271 ppb bias and 442 ppb precision), also outperforming the state‐of‐the‐art acetone sensors (e.g., adsorption column,^[^ ^18^ ^]^ enzymatic sensor,^[^ ^28^ ^]^ and a sensor array comprising a SnO_2_ and a Pt/WO_3_ sensor,^[^ ^22^ ^]^ Figure 6d–f) as well as commercial devices (e.g., LEVL,^[^ ^69^ ^]^ Table 1). These detectors consistently overpredicted acetone concentrations (i.e., bias >132 ppb), suggesting sensitivity to interferants. Only a single‐use (disposable) colorimetric sensor^[^ ^70^ ^]^ achieved a better bias (i.e., 12 ppb, Figure 6c) but worse precision (i.e., 626 ppb). It should be noted that the adsorption column^[^ ^18^ ^]^ and the SnO_2_ and Pt/WO_3_^[^ ^22^ ^]^ sensor array were tested during dieting (leading to larger breath acetone ranges and possibly other interferants), whereas the testing protocols for the colorimetric^[^ ^70^ ^]^ and enzymatic sensor^[^ ^28^ ^]^ had not been specified.

Finally, it is important to highlight that the BOHB concentrations (measured here in a clinical laboratory) ranged from 0.04 to 0.74 mmol L^−1^ (Figure S5, Supporting Information), whereas the commercial capillary blood ketone strips (e.g., FreeStyle and Abbott) are limited typically by a mean bias of 0.1 mmol L^−1^.^[^ ^71^ ^]^ Thus, they may fail to resolve such fine lipolysis differences, particularly induced by exercise (0 ≤ *t* < 60 min, blood BOHB 0.04–0.22 mmol L^−1^, Figure S5, Supporting Information). This highlights even more the potential of point‐of‐care and non‐invasive breath acetone monitoring with highly accurate detectors for mHealth applications. The present detector is light, small (e.g., filter [30 mg powder, 1.5 cm length × 4 mm diameter] and sensor [15 mm × 13 mm × 0.8 mm]), and reusable, showing good stability over, at least, 20 days (Figure S7, Supporting Information), making it promising for integration into a handheld and smartphone‐assisted device^[^ ^72^ ^]^ to guide exercising and dieting, as with breath methanol.^[^ ^73^ ^]^

## 3 Conclusion

A low‐cost detector was presented for robust breath acetone monitoring, a non‐invasive indicator of lipolysis during exercise and rest. Accurate ppb‐level acetone quantification was enabled even in short (5 s) exhalations by screening a chemoresistive Si/WO_3_ sensor with a modular catalytic Pt/Al_2_O_3_ filter that removed critical isoprene and ethanol that either spiked during exercise or had been released during disinfection. Even fine metabolic acetone changes during a cardiorespiratory fitness‐adapted exercise protocol and subsequent resting were traced in nine volunteers, in good agreement with PTR‐ToF‐MS and validated by venous blood BOHB measurements. In fact, excellent bias and unprecedented precision of 25 and 169 ppb, respectively, were achieved, superior to state‐of‐the‐art acetone detectors. This is required to guide personalized exercise and diets, where small acetone changes (e.g., <100 ppb) need to be resolved. As a result, this detector is most promising as an mHealth device for metabolic monitoring. Due to its modular design, it can be flexibly integrated into handheld devices for widespread use.

## 4 Experimental Section

4.1

4.1.1

##### Study Protocol

The study includes nine healthy volunteers (four female) with age and height provided in Table S1, Supporting Information. All volunteers were free of known cardiovascular, respiratory or metabolic diseases, non‐smoking, and did not follow special diets (e.g., low carb). In addition, all volunteers were asked to abstain from alcohol and intensive exercise 24 h prior to the study appointments.

The first appointment served to determine the VO_2peak_ and the workload at the second ventilatory threshold (VT_2_, Ergoline ErgoSelect 200, Germany equipped with MetaLyzer 3B‐R2 spirometer, Cortex Biophysik GmbH, Germany) during an exhaustive spiroergometry test, both being indicators of individual cardiorespiratory fitness.^[^ ^34^ ^]^ The VO_2peak_ is a measure of the maximum O_2_ uptake^[^ ^74^ ^]^ and was calculated by taking an average of the three highest values obtained. Female and male participants with a VO_2peak_ > 34 and 40 mL kg^−1^ min^−1^, respectively, were classified as high fit, in agreement with the literature.^[^ ^62^ ^]^ The VT_2_ is the intensity threshold at which the body changes from the aerobic to anaerobic metabolism^[^ ^75^ ^]^ and was determined by experienced physicians as the point where ventilation rates exceeded VO_2_ rates. In addition, the body weight was measured (InBody720, InBody Co., Ltd., South Korea).

The second appointment took place, at least, five days to, maximum, three weeks after the first appointment, to ensure that volunteers recovered fully from the initial workload. On the evening before the second appointment, a low‐carb dinner was consumed. The study started at 8 am the following morning (after overnight fasting), and volunteers were instructed not to use chemical mouthwash, at least, 2 h before 8 am. A submaximal exercise protocol standardized to the individual cardiorespiratory fitness was applied.^[^ ^34^ ^]^ The graded exercise protocol started at 20% of the individual VT_2_ and increased up to 100% VT_2_, with increasing 10% VT_2_ steps every 5 min (Figure 3a). Once 100% VT_2_ was reached (i.e., after 40 min), the volunteers were encouraged to continue until exhaustion or for a maximum of 15 min. Throughout the exercise, the volunteers maintained a cadence of around 70 rpm. This study was approved by the Ethikkommission Nordwest‐ und Zentralschweiz (EKNZ 2019‐02362). Prior to the study, all participants gave informed written consent.

##### Breath and Blood Sampling

Breath samples (Figure 3a, stars) were collected before the exercise start, during exercise (i.e., at the end of each increment) and during 3 h of subsequent rest (every 30 min). Breath sampling was done with an inert and heated (i.e., 60 °C) buffered end‐tidal sampler comprising a disposable mouthpiece and an open‐ended sampling tube with a tube volume of 270 mL and no flow restrictor, to allow for fast exhalations during exercise.^[^ ^58^ ^]^ Participants were asked to exhale completely within 5 s through a sterile and removable mouthpiece (EnviteC‐Wismar GmbH, Germany) that results in reproducible end‐tidal breath acetone concentrations, as confirmed by a PTR‐ToF‐MS 1000 (Ionicon, Austria). A CO_2_ sensor (Capnostat 5, Respironics, USA) was used to monitor breath CO_2_.

Blood samples (Figure 3a, diamonds) were taken before and immediately after exercise, as well as at 80, 110, 170, and 230 min afterward. Blood sampling was done through an intravenous^[^ ^43^ ^]^ Venflon line that was installed in the morning prior to measurements. Before each blood sampling, a hand disinfectant (80 wt% ethanol with 1% glycerin, B. Braun Medical AG) was used. All samples contained 7.5 mL blood and were centrifuged for 10 min (3000 rpm, 20 °C; Universal 320R, Hettich Zentrifugen, Switzerland) immediately after collection to separate serum from plasma. The serum was stored at −80 °C and analyzed at the end of the study to determine BOHB (Institute of Clinical Chemistry University Hospital Zurich, Switzerland). Note that for BOHB levels below 0.1 mmol L^−1^, quantification is less accurate.

##### Breath Analysis

The breath samples were analyzed with the acetone detector and PTR‐ToF‐MS (before the filter) drawing 150 mL min^−1^ from the breath sampler with a vane pump (Schwarzer Precision, Germany). Inert and heated Teflon tubing was used to avoid analyte adsorption and water condensation. The acetone detector comprised a compact, tubular, catalytic packed bed filter^[^ ^76^ ^]^ at 135 °C.^[^ ^41^ ^]^ Downstream of the filter, a chemoresistive sensor quantified the acetone concentration. The sensor was made of flame‐deposited and in situ annealed 10 mol% Si/WO_3_ nanoparticles^[^ ^23^ ^]^ on interdigitated Pt electrodes on Al_2_O_3_ substrates in a Teflon chamber^[^ ^45^ ^]^ and heated to 400 °C.

The sensor resistance was determined using a multimeter (Keithley 2700, USA), and its response was calculated as *R*_air_/*R*_breath_ − 1, with *R*_air_ the resistance in room air, and *R*_breath_ the minimum resistance during breath exposure. The response time was determined as the time needed to reach 90% of the sensor response. Prior to the study, a five‐point acetone calibration was carried out and repeated on each measurement day with 1 ppm to assess the filter stability. For this, synthetic gas mixtures containing the calibrated gas standard (18 ppm acetone in synthetic air, Pan Gas, Switzerland, C_*n*_H_*m*_ and NO_*x*_ ≤ 100 ppb) at 90% RH were prepared with a high‐resolution mixing setup^[^ ^77^ ^]^ into Tedlar bags (3L, SKC Inc., USA). These Tedlar bags were connected to the analysis unit through Teflon tubing and drawn to the sensor by the vane pump (e.g., 150 mL min^−1^). For breath acetone quantification, values were compared with the linear regression of the calibration (Figure S3, Supporting Information).

The PTR‐ToF‐MS was operated at 600 V, 2.3 mbar, and 60 °C with H_3_O^+^ as primary ions. Analyte concentrations were determined at the mass‐to‐charge (m/z) ratios of 33.03 (methanol^[^ ^78^ ^]^), 47.05 (ethanol^[^ ^79^ ^]^), 59.05 (acetone^[^ ^79^ ^]^), 61.05 (2‐propanol^[^ ^80^ ^]^), and 69.07 (isoprene^[^ ^78^ ^]^). Prior to measurements, five‐point calibrations over the relevant range were carried out using calibrated gas standards (all Pangas, in synthetic air as mentioned earlier) for methanol (20 ppm), ethanol (10 ppm), acetone (18 ppm), 2‐propanol (200 ppm), and isoprene (16 ppm).

##### Material Characterization

The Pt/Al_2_O_3_ nanoparticles were visualized with an aberration‐corrected scanning transmission electron microscope (HD‐2700CS, Hitachi, Japan) at 200 kV, equipped with a secondary electron and a high angle annular dark field detector. Prior to imaging, the nanoparticles were suspended in ethanol, sonicated, and subsequently deposited onto a perforated carbon foil supported on a copper grid. To investigate the cross sections of the Si/WO_3_ chemoresistive sensing films, the sensors were split and subsequently imaged by scanning electron microscopy with a Hitachi field emission scanning electron microscope 4000 operated at 5 kV.

##### Statistical Analysis

No data pre‐processing was done. For experimental measurements repeated under the same conditions (including, at least, three replicates), mean ± *σ* was calculated. The sample sizes (*n*) for each statistical analysis are indicated in the figure legends. Comparison between blood and breath data was assessed by calculating Spearman's (*r*_s_) and Pearson's (*r*_p_) correlation coefficients, and statistical significance was evaluated with independent two‐sampled *t*‐tests. Linear fits are provided together with their coefficients and coefficient of determination (*R* ^2^) in Figure 5. Statistical Bland–Altman analysis^[^ ^46^ ^]^ (in Figure 6) was carried out to assess the agreement between sensor and PTR‐ToF‐MS, as standard in medical diagnostics when comparing new devices with “gold standard” methods. Therein, the bias^[^ ^81^ ^]^ (i.e., estimate of a systematic measurement error) corresponded to the average difference between sensor and MS or GC technology, whereas the precision was defined as 2*σ* due to the large number of samples (i.e., ≥38).^[^ ^82^ ^]^ Replotting of literature data (i.e., in Figure 6) was done with the program WebPlotDigitizer. For statistical analyses, the software OriginPro 2018G (OriginLab Corporation, USA) was used.

## Conflict of Interest

The authors declare no conflict of interest.

## Data Availability Statement

The data that supports the findings of this study are available in the supplementary material of this article.

## Supporting information

Supplementary Material
