# Supplementary material for: Monitoring Lipolysis by Sensing Breath Acetone down to Parts‐per‐Billion
Source: Small Sci. 2021 Mar 12;1(4):2100004. doi: 10.1002/smsc.202100004 (PMC11935798; doi:10.1002/smsc.202100004)
Supplement: Supplementary file 1 — Supplementary Material [file SMSC-1-2100004-s001.pdf]

Copyright WILEY-VCH Verlag GmbH & Co. KGaA, 69469 Weinheim, Germany, 2018.

Supporting Information

Monitoring Lipolysis by Sensing Breath Acetone down to ppb

Ines C. Weber, Nina Derron, Karsten Königstein, Philipp A. Gerber, Andreas T. Güntner, Sotiris E. Pratsinis*

Figures & Captions

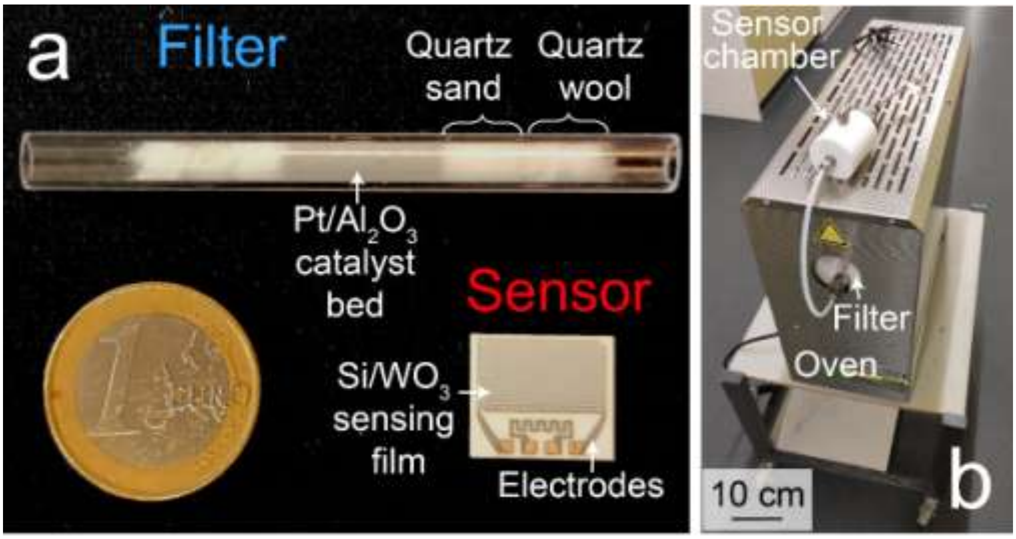

Figure S1. (a) Photograph of the packed bed Pt/Al_2O_3 catalytic filter sandwiched by quartz wool and sand from both sides together with the Si/WO_3 sensor in comparison to a 1-Euro coin. (b) Photograph of the assembled detector comprising that filter (packed bed inside an oven) connected with inert Teflon tubing to the chamber containing the sensor.

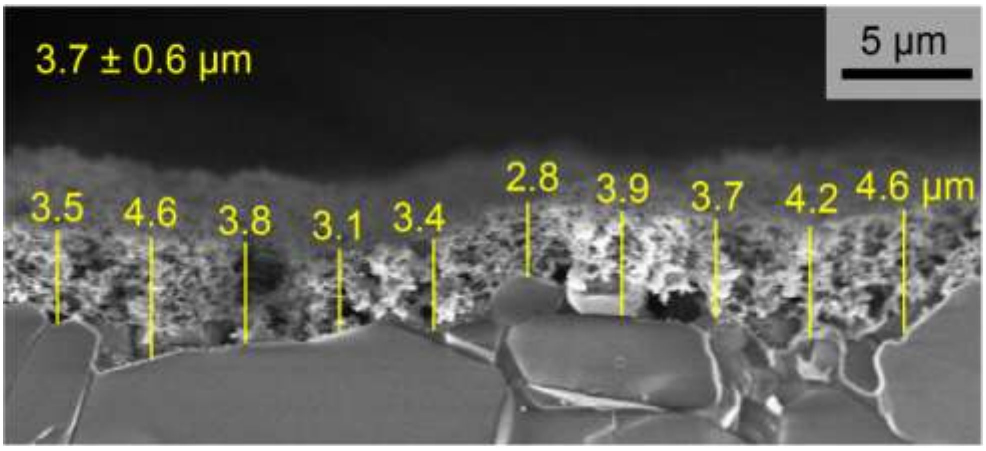

Figure S2. Si/WO_3 sensing film thicknesses (n = 10) at designated locations are depicted. Average thickness ± σ is indicated at upper left.

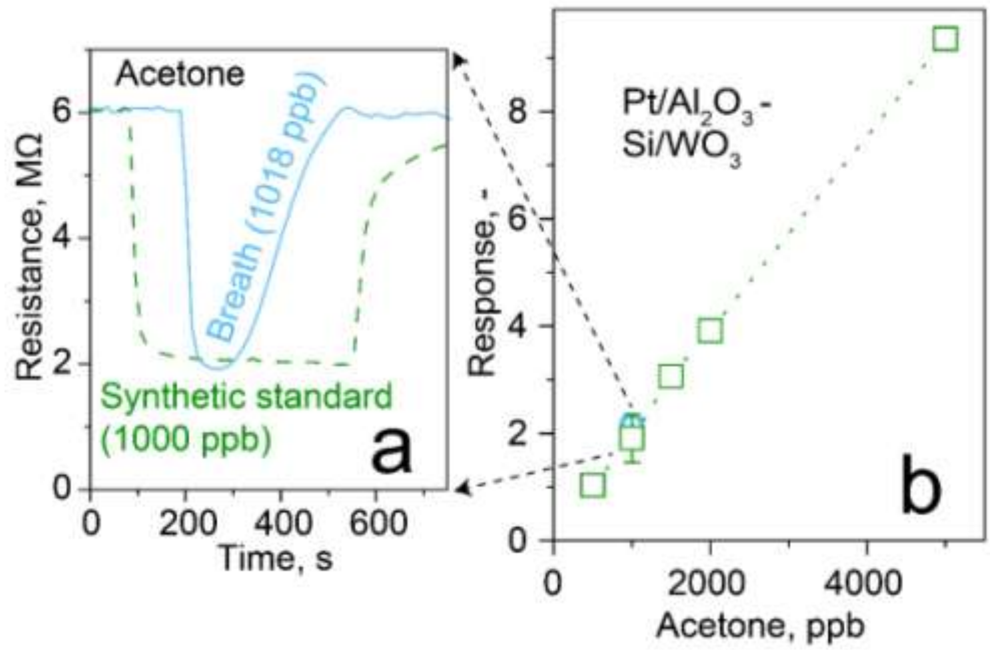

Figure S3. (a) Detector resistance change when exposed to 1000 ppb acetone in synthetic air at 90% RH (dashed line) and 1018 ppb acetone from real breath (solid line). Note that different exposure times had to be applied due to the physiologically limited duration of an exhalation (i.e., 5 s). More such film resistance graphs for 50 – 1000 ppb acetone alone in synthetic air and in gas mixtures with ammonia, isoprene, ethanol, CO and H_2 at 50% RH are provided for the same detector elsewhere.[41] There, we had even compared it directly to PTR-ToF-MS signals. (b) Detector response to acetone in synthetic air at 90% RH (squares) and this breath sample (circle). The error bar at 1000 ppb represents n = 9 measurements where the synthetic mixtures were obtained on separate days. Note that the Pt/Al_2O_3 - Si/WO_3 detector calibration accounted for the 23% concentration-independent[41] acetone loss within the catalytic filter.

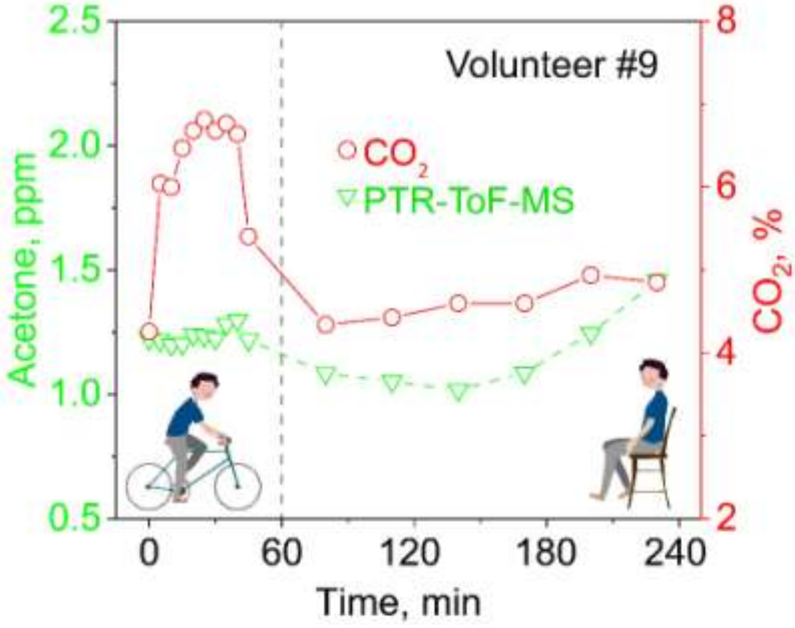

Figure S4. Breath acetone (triangles, left ordinate) and CO_2 (circles, right ordinate) concentrations for volunteer #9 during exercise (t < 60 min) and rest (t > 60 min).

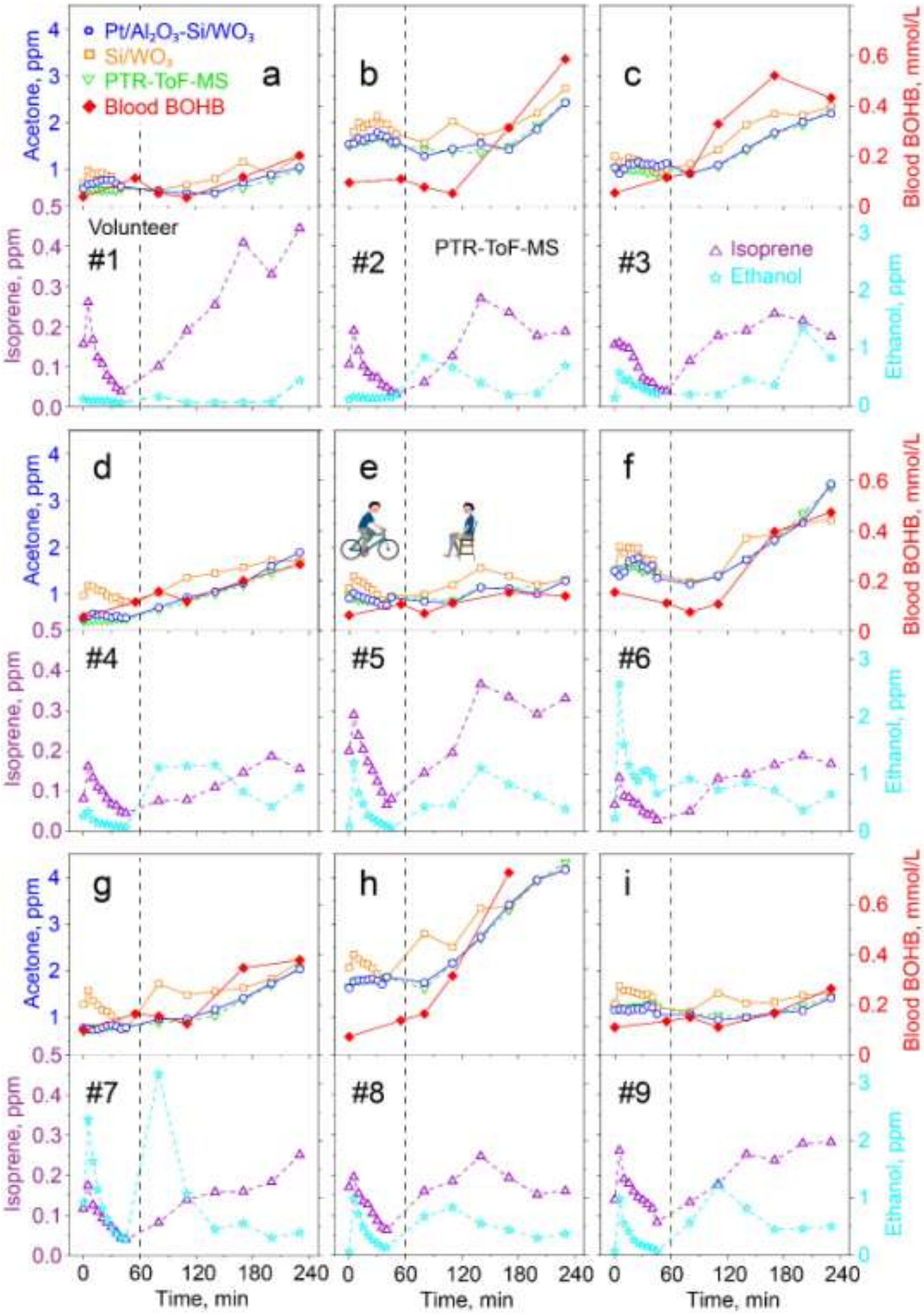

Figure S5. Breath acetone (left ordinate) as measured by PTR-ToF-MS (inverted triangles) and the Si/WO_3 sensor with (circles) or without (squares) the Pt/Al_2O_3 filter together with venous blood BOHB measurements (right ordinate, diamonds) for volunteers #1 - #9 during exercise (t < 60 min) and rest (t > 60 min). Note that due to blood clotting, the BOHB of volunteer #8 at 230 min is missing. Additionally, interfering breath isoprene (left ordinate, triangles) and ethanol concentrations (right ordinate, stars) by the PTR-ToF-MS are shown.

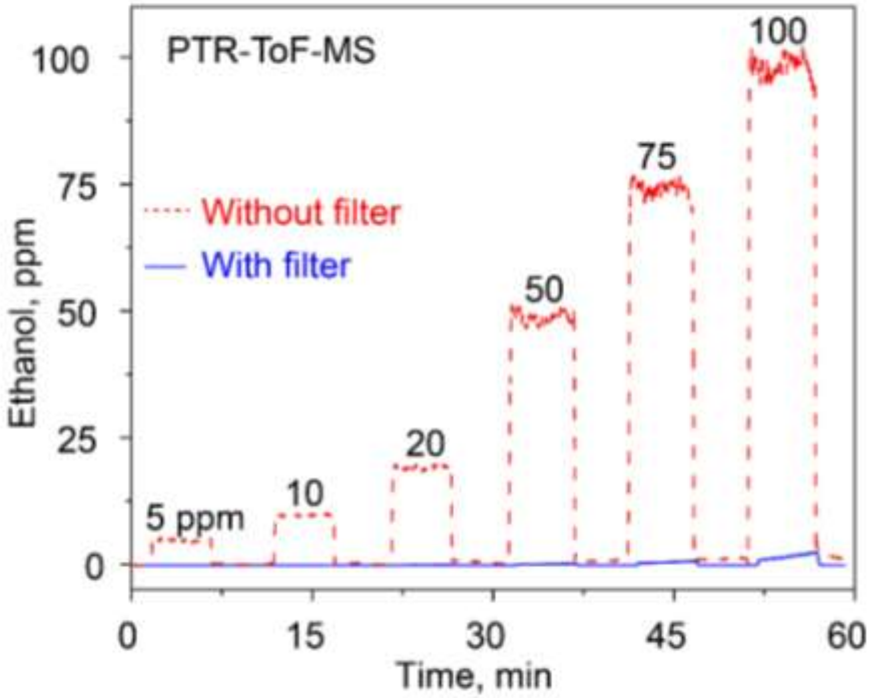

Figure S6. Synthetic gas mixtures containing 5 – 100 ppm ethanol in 50% RH, as detected by the PTR-ToF-MS without (red, dotted line) and with (blue, solid line) the Pt/Al_2O_3 filter.

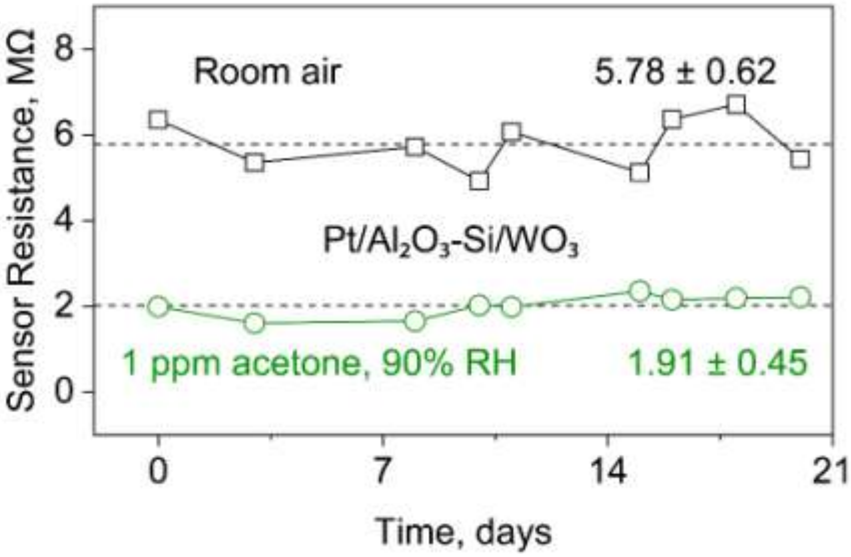

Figure S7. Baseline resistance in room air (squares), as well as resistance to 1 ppm acetone at 90% RH (circles). The Si/WO_3 sensor and Pt/Al_2O_3 filter were operated continuously for 20 days.

Table S1. Age, gender, height, weight, BMI and weight-adjusted VO_{2peak} of all volunteers.

ID [-]	Age [y]	Gender [-]	Height [cm]	Weight [kg]	BMI [kg/m^2]	Weight-adjusted VO_{2peak} [mL/kg/min]
1	26	f	164	56.1	20.9	60
2	25	f	169	53.5	18.8	46
3	27	m	173	76.6	25.6	41
4	24	f	165	51.0	18.7	33
5	22	m	189.8	101.6	28.2	40
6	24	m	182	64.1	19.4	51
7	23	f	160	55.2	19.7	39
8	24	m	171	63.2	21.6	45
9	22	m	191	105.5	24.1	39
